# Classification of major depressive disorder using vertex-wise brain sulcal depth, curvature, and thickness with a deep and a shallow learning model

**DOI:** 10.1038/s41380-025-03273-w

**Published:** 2025-10-03

**Authors:** Roberto Goya-Maldonado, Tracy Erwin-Grabner, Ling-Li Zeng, Christopher R. K. Ching, Andre Aleman, Alyssa R. Amod, Zeynep Basgoze, Francesco Benedetti, Bianca Besteher, Katharina Brosch, Robin Bülow, Romain Colle, Colm G. Connolly, Emmanuelle Corruble, Baptiste Couvy-Duchesne, Kathryn Cullen, Udo Dannlowski, Christopher G. Davey, Annemiek Dols, Jan Ernsting, Jennifer W. Evans, Lukas Fisch, Paola Fuentes-Claramonte, Ali Saffet Gonul, Ian H. Gotlib, Hans J. Grabe, Nynke A. Groenewold, Dominik Grotegerd, Tim Hahn, J. Paul Hamilton, Laura K. M. Han, Ben J. Harrison, Tiffany C. Ho, Neda Jahanshad, Alec J. Jamieson, Andriana Karuk, Tilo Kircher, Bonnie Klimes-Dougan, Sheri-Michelle Koopowitz, Thomas Lancaster, Ramona Leenings, Meng Li, David E. J. Linden, Frank P. MacMaster, David M. A. Mehler, Susanne Meinert, Elisa Melloni, Bryon A. Mueller, Benson Mwangi, Igor Nenadić, Amar Ojha, Yasumasa Okamoto, Mardien L. Oudega, Brenda W. J. H. Penninx, Sara Poletti, Edith Pomarol-Clotet, Maria J. Portella, Joaquim Radua, Elena Rodríguez-Cano, Matthew D. Sacchet, Raymond Salvador, Anouk Schrantee, Kang Sim, Jair C. Soares, Aleix Solanes, Dan J. Stein, Frederike Stein, Aleks Stolicyn, Sophia I. Thomopoulos, Yara J. Toenders, Aslihan Uyar-Demir, Eduard Vieta, Yolanda Vives-Gilabert, Henry Völzke, Martin Walter, Heather C. Whalley, Sarah Whittle, Nils Winter, Katharina Wittfeld, Margaret J. Wright, Mon-Ju Wu, Tony T. Yang, Carlos Zarate, Dick J. Veltman, Lianne Schmaal, Paul M. Thompson

**Affiliations:** 1 Laboratory of Systems Neuroscience and Imaging in Psychiatry (SNIP-Lab), Department of Psychiatry and Psychotherapy, University Medical Center Göttingen (UMG), Georg-August University, Göttingen, Germany; 2 College of Intelligence Science and Technology, National University of Defense Technology, Changsha, 410073 China; 3 Imaging Genetics Center, Mark & Mary Stevens Neuroimaging and Informatics Institute, Keck School of Medicine, University of Southern California, Marina del Rey, CA 90274 USA; 4 Department of Biomedical Sciences of Cells and Systems, University Medical Center Groningen, University of Groningen, Groningen, the Netherlands; 5 Department of Psychiatry & Mental Health, Neuroscience Institute, University of Cape Town, Cape Town, South Africa; 6 Department of Psychiatry and Behavioral Science, University of Minnesota Medical School, Minneapolis, MN USA; 7 Division of Neuroscience, IRCCS Scientific Institute Ospedale San Raffaele, Milano, Italy; 8 Department of Psychiatry and Psychotherapy, Jena University Hospital, Jena, Germany; 9 Department of Psychiatry and Psychotherapy, University of Marburg, Rudolf Bultmann Str. 8, 35039 Marburg, Germany; 10 Institute for Radiology and Neuroradiology, University Medicine Greifswald, Greifswald, Germany; 11 MOODS Team, CESP, INSERM U1018, Faculté de Médecine, Univ Paris-Saclay, Le Kremlin Bicêtre, 94275 France; 12 Service Hospitalo-Universitaire de Psychiatrie de Bicêtre, Hôpitaux Universitaires Paris-Saclay, Assistance Publique-Hôpitaux de Paris, Hôpital de Bicêtre, Le Kremlin Bicêtre, F-94275 France; 13 Department of Biomedical Sciences, Florida State University, Tallahassee, FL USA; 14 Sorbonne University, Paris Brain Institute - ICM, CNRS, Inria, Inserm, AP-HP, Hôpital de la Pitié Salpêtrière, F-75013 Paris, France; 15 Institute for Molecular Bioscience, the University of Queensland, St Lucia, QLD Australia; 16 Institute for Translational Psychiatry, University of Münster, Münster, Germany; 17 Melbourne Neuropsychiatry Centre, Department of Psychiatry, the University of Melbourne, Parkville, VIC Australia; 18 Department of Psychiatry, Amsterdam UMC, Vrije Universiteit Amsterdam, Amsterdam Neuroscience, Amsterdam Public Health Research Institute, Amsterdam, the Netherlands; 19 Department of Psychiatry, UMC Utrecht Brain Center, University Utrecht, Utrecht, the Netherlands; 20 Experimental Therapeutics and Pathophysiology Branch, National Institute for Mental Health, National Institutes of Health, Bethesda, MD USA; 21 FIDMAG Germanes Hospitalàries Research Foundation, Centro de Investigación Biomédica en Red de Salud Mental (CIBERSAM), Instituto de Salud Carlos III, Barcelona, Catalonia Spain; 22 SoCAT Lab, Department of Psychiatry, School of Medicine, Ege University, Izmir, Turkey; 23 Department of Psychology, Stanford University, Stanford, CA USA; 24 Department of Psychiatry and Psychotherapy, University Medicine Greifswald, Greifswald, Germany; 25 Center for Social and Affective Neuroscience, Department of Biomedical and Clinical Sciences, Linköping University, Linköping, Sweden; 26 Centre for Youth Mental Health, the University of Melbourne, Parkville, VIC Australia; 27 Orygen, Parkville, VIC Australia; 28 Department of Psychiatry and Behavioral Sciences, Division of Child and Adolescent Psychiatry, Weill Institute for Neurosciences, University of California, San Francisco, CA USA; 29 Department of Psychology, University of California, Los Angeles, CA USA; 30 Department of Psychology, University of Minnesota, Minneapolis, MN USA; 31 Cardiff University Brain Research Imaging Centre, Cardiff University, Cardiff, UK; 32 MRC Centre for Neuropsychiatric Genetics and Genomics, Cardiff University, Cardiff, UK; 33 Division of Psychological Medicine and Clinical Neurosciences, Cardiff University, Cardiff, UK; 34 School of Mental Health and Neuroscience, Faculty of Health, Medicine and Life Sciences, Maastricht University, Maastricht, 6229 ER the Netherlands; 35 Departments of Psychiatry and Pediatrics, University of Calgary, Calgary, AB Canada; 36 Department of Psychiatry, Psychotherapy and Psychosomatics, Medical School, RWTH Aachen University, Aachen, Germany; 37 Institute for Translational Neuroscience, University of Münster, Münster, Germany; 38 Center Of Excellence on Mood Disorders, Louis A. Faillace, MD, Department of Psychiatry and Behavioral Sciences at McGovern Medical School, the University of Texas Health Science Center at Houston, Houston, TX USA; 39 Center for Neuroscience, University of Pittsburgh, Pittsburgh, PA USA; 40 Center for Neural Basis of Cognition, University of Pittsburgh, Pittsburgh, PA USA; 41 Department of Psychiatry and Neurosciences, Hiroshima University, Hiroshima, Japan; 42 GGZ inGeest Mental Health Care, Amsterdam, the Netherlands; 43 Sant Pau Mental Health Research Group, Institut de Recerca de l’Hospital de la Santa Creu i Sant Pau, Barcelona, Catalonia Spain; 44 CIBERSAM, Madrid, Spain; 45 Imaging of Mood- and Anxiety-Related Disorders (IMARD) Group, Institut d’Investigacions Biomèdiques August Pi i Sunyer (IDIBAPS), Barcelona, Spain; 46 Meditation Research Program, Department of Psychiatry, Massachusetts General Hospital, Harvard Medical School, Boston, MA USA; 47 Amsterdam University Medical Centers, location AMC, Department of Radiology and Nuclear Medicine, Amsterdam, the Netherlands; 48 West Region, Institute of Mental Health, Singapore, Singapore; 49 Yong Loo Lin School of Medicine, National University of Singapore, Singapore, Singapore; 50 Lee Kong Chian School of Medicine, Nanyang Technological University, Singapore, Singapore; 51 Division of Psychiatry, Centre for Clinical Brain Sciences, University of Edinburgh, Scotland, UK; 52 Developmental and Educational Psychology, Leiden University, Leiden, the Netherlands; 53 Erasmus School of Social and Behavioral Sciences, Erasmus University Rotterdam, Rotterdam, the Netherlands; 54 Hospital Clinic, Institute of Neuroscience, University of Barcelona, IDIBAPS, CIBERSAM, Barcelona, Catalonia Spain; 55 Intelligent Data Analysis Laboratory (IDAL), Department of Electronic Engineering, Universitat de València, Valencia, Spain; 56 Institute for Community Medicine, University Medicine Greifswald, Greifswald, Germany; 57 Queensland Brain Institute, the University of Queensland, Brisbane, QLD Australia; 58 Centre for Advanced Imaging, the University of Queensland, Brisbane, QLD Australia; 59 Section on the Neurobiology and Treatment of Mood Disorders, National Institute of Mental Health, Bethesda, MD USA

**Keywords:** Depression, Neuroscience

## Abstract

Major depressive disorder (MDD) is a complex psychiatric disorder that affects the lives of hundreds of millions of individuals around the globe. Even today, researchers debate if morphological alterations in the brain are linked to MDD, likely due to the heterogeneity of this disorder. The application of deep learning tools to neuroimaging data, capable of capturing complex non-linear patterns, has the potential to provide diagnostic and predictive biomarkers for MDD. However, previous attempts to demarcate MDD patients and healthy controls (HC) based on segmented cortical features via linear machine learning approaches have reported low accuracies. In this study, we used globally representative data from the ENIGMA-MDD working group containing 7012 participants from 31 sites (N = 2772 MDD and N = 4240 HC), which allows a comprehensive analysis with generalizable results. Based on the hypothesis that integration of vertex-wise cortical features can improve classification performance, we evaluated the classification of a DenseNet and a Support Vector Machine (SVM), with the expectation that the former would outperform the latter. As we analyzed a multi-site sample, we additionally applied the ComBat harmonization tool to remove potential nuisance effects of site. We found that both classifiers exhibited close to chance performance (balanced accuracy DenseNet: 51%; SVM: 53%), when estimated on unseen sites. Slightly higher classification performance (balanced accuracy DenseNet: 58%; SVM: 55%) was found when the cross-validation folds contained subjects from all sites, indicating site effect. In conclusion, the integration of vertex-wise morphometric features and the use of the non-linear classifier did not lead to the differentiability between MDD and HC. Our results support the notion that MDD classification on this combination of features and classifiers is unfeasible. Future studies are needed to determine whether more sophisticated integration of information from other MRI modalities such as fMRI and DWI will lead to a higher performance in this diagnostic task.

## Introduction

Major depressive disorder (MDD) dramatically impacts the daily functioning of patients and is currently the leading cause of disability worldwide [1]. Therefore, early diagnosis and optimal allocation of the proper treatment are critical. Unfortunately, the current treatment strategies present a response rate and remission as low as of 36.8% after a first treatment [2–4]. Thus, as proposed in the realms of systems medicine, we expect that by identifying brain patterns that classify patients at the individual level, we may open new biomarker-based avenues for the development of more personalized and effective treatments.

Neuroimaging techniques, such as magnetic resonance imaging (MRI), enable a non-invasive macro-scale view of human brain structure at the millimeter level of resolution. Initial neuroimaging studies used univariate approaches to reveal structural brain differences in MDD compared to healthy controls (HC) [5–7], identifying reduced hippocampal and frontal lobe volume. However, these studies had limited sample sizes and the more recent large sample studies have reported small effect sizes [8–11], highlighting the absence of a single neuro-anatomical biomarker associated with MDD. The search for more complex biomarkers, which may include the interaction between different neuro-anatomical features, can be conducted via machine learning (ML) algorithms - especially deep learning (DL) algorithms - applied to the MDD vs HC classification task.

Like univariate approaches, ML and DL studies reported varying classification accuracies from 53–91% [12, 13]. The high variability of classification performances and the lack of consistent biomarkers can partly be explained by the small sample sizes, as it was demonstrated by Flint and colleagues [14]. Supplementing this, a study based on cortical and subcortical morphological features, reported high accuracy of 75% in the small sample, which was not replicated in an independent large UK Biobank dataset, achieving only 54% [15].

Another factor that may inflate classification accuracies are related to study-site effects. The site-effect corresponds to site-specific characteristics other than diagnosis – such as scanner type, acquisition protocol, demographic differences, and inclusion and exclusion criteria – which may bias classification accuracies. A study demonstrated how site effect may contribute to both inflated and deflated classification accuracies [16]. Hence, numerous ways to tackle site-effect and improve model generalizability exist, from linear and non-linear ComBat harmonization tools [17, 18] to embedding site confounders directly to the model [19]. However, to overcome the difficult point of the heterogeneity of MDD and the lack of replicability and generalization of the models, the investigation of very large samples of participants with global representation is fundamental.

Using a large-scale dataset from the ENIGMA-MDD consortium, we compared the classification performance of commonly used ML models to predict diagnosis based on cortical and subcortical parcellations of morphological features (surface areas, thicknesses, volumes) [20]. Overall, results showed a trend that may highlight the contribution of site-effects to classification performance. Specifically, there was a clear difference in classification performance dependent on the cross-validation splitting techniques used in training. Site-splitting generally performed at close to chance level for all classifiers, while mixing sites across splits achieved up to 62% balanced accuracy with an SVM. Of note, data harmonization using ComBat removed the site effect and resulted in a balanced accuracy of 52% with SVM. Based on these findings, we concluded that most commonly used ML classification algorithms could not successfully discriminate MDD from HC individuals based on morphological features organized in pre-defined Desikan-Killiany atlas parcellations. However, it remains unclear whether more fine-grained information of morphometric features, displayed in a vertex-wise organization, could outperform the classification based on parcellation atlas-distributed information.

There are some directions in improving classification based on morphological information. First, previous ML studies considered surface area, thickness, and volume characteristics only, while the information on the cortical shape, such as gyral and sulcal shape patterns, was not integrated into analyses. Cortical gyrification modalities are affected by genetic and non-genetic factors [21, 22], alterations of which were associated with MDD [23, 24]. Multimodal morphological feature analysis, including myelination, gray matter, and curvature, revealed a correlation between cortical differences and MDD-associated genes [25]. Therefore, the addition of shape modalities, such as cortical curvature and sulcal depth, to cortical thickness could enhance the classification performance, as demonstrated for sex and autism classification [26].

Cortical morphological features such as sulcal depth and gyrification, measured via local gyrification index (LGI) or curvature, have been investigated as potential biomarkers for MDD, although the literature remains limited and somewhat inconsistent. Some earlier studies have suggested that sulcal depth may be decreased in individuals with suicidality-associated MDD [25]. Even so, this study included only 39 healthy controls, 40 depressed patients without suicidality (patient controls), and 39 with suicidality (suicidal groups) were analyzed based on SBM to estimate the fractal dimension, gyrification index, sulcal depth, and cortical thickness; the small sample size and range of features assessed make it prone to both type I and type II error, relative to the studies we have performed in thousands of patients. In terms of gyrification, multiple studies have reported both hypo- and hyper-gyrification in various cortical regions, including the frontal, cingulate, insular, parietal, and temporal lobes [24, 27–31]. However, these findings are often region-constrained, based on small sample sizes, and lack consistent replication across cohorts and studies. These constraints highlight the need for coordinated multi-site analyses using harmonized data and advanced morphometric modeling approaches.

Hence, one promising direction is the use of more advanced classification algorithms. DL methods have gained popularity in the neuroimaging field as a promising tool for cortical surface reconstruction [32], image preprocessing [33], and cortical parcellation [34]. Furthermore, DL is widely evaluated as a predictive tool in psychiatry, showing higher or at least the same classification performance compared to linear models [26, 35–39]. The analysis of cortical morphometric features can be conducted via convolutional neural network (CNN) [40], designed to reveal complex patterns in 2D images. In order to apply such 2D CNN in the classification, it requires 3D cortical features to be initially projected into 2D image space. Nevertheless, this step may inevitably create distortion in spatial properties such as shape, area, distance, and direction. Several approaches were implemented before, such as latitude/longitude projection [41] and optimal mass transport (OMT) projection [26, 42], which preserves area. However, the impact of these projection methods on classification performance were never directly compared in the neuroimaging field.

The main goal of this study was to distinguish MDD from HC individuals based on integrated cortical morphological features, including sulcal depth, curvature, and thickness. These features were analyzed via SVM with linear kernel and CNN architecture of pre-trained DenseNet [43], which demonstrated its superiority over simpler models in autism vs HC classification task [26]. SVM was chosen as it is a robust shallow ML model, frequently used in neuroimaging settings [44–46]. We investigated classification performance of these two methods to understand the role of complex non-linear patterns in MDD manifestation. We used balanced accuracy, sensitivity, specificity and AUC as the classification performance metrics. Higher classification performance of the DenseNet model presume the presence of spatially complex patterns in brain morphology, which are relevant for classification. Furthermore, we aimed to estimate the relevance of integrating cortical thickness and shape characteristics (sulcal depth, curvature and thickness) into the analysis by training the models with all features combined and by considering them separately. Similar to our previous study [20], different cross-validation (CV) approaches were evaluated: Splitting the data by balancing age and sex distribution across all CV folds (Splitting by Age/Sex), and performing leave-sites-out CV in order to estimate the performance on the unseen during the training sites (Splitting by Site). This approach allowed us to estimate whether the model’s performance is influenced by demographic or site-related factors. The difference between results in both splitting strategies presumes the presence of the site effect we addressed by harmonizing the data in both splitting strategies via ComBat. In summary, we hypothesized that: (1) Integration of cortical thickness and shape characteristics would contribute positively to the classification performance, and (2) DenseNet could differentiate MDD from HC based on the provided features. Additionally, we compared two projection methods, latitude/longitude and OMT projections by performing auxiliary single-site sex classification based on three of the largest cohorts to explore whether classification performance may vary according to 2D projection method. We had no a priori hypothesis for the projection results.

## Material and methods

### Study participants and study design

We analyzed a large-scale multi-site sample provided by the ENIGMA-MDD working group, comprising 2772 MDD and 4240 HC individuals, from 30 cohorts worldwide. Details on inclusion/exclusion criteria and sample characteristics can be found in Supplementary Table 1. Subjects with missing information on demographic data or any of cortical surface mesh files (l(r).sulc, l(r).curv, l(r).thickness) were excluded from the analysis (476 and 6% excluded).

### Image processing and analysis

**Fig. 1:**
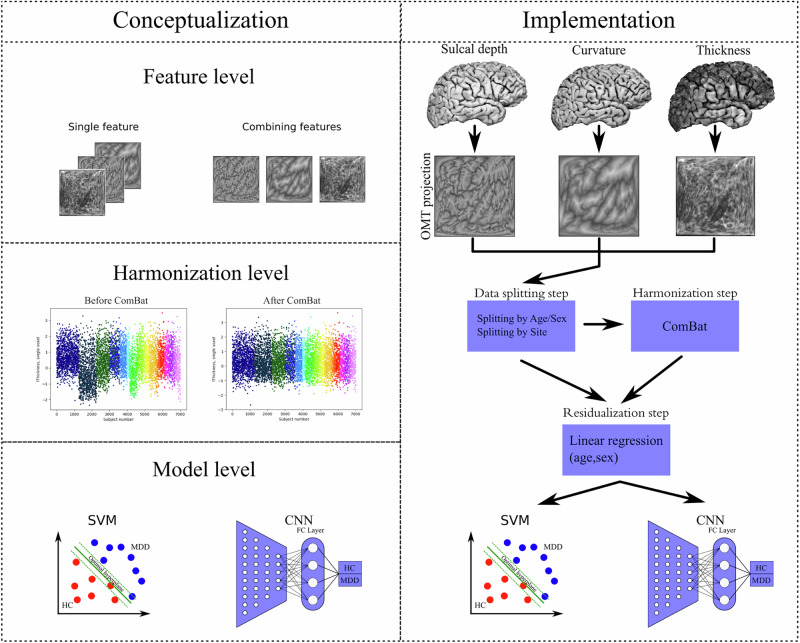
Proposed conceptualization levels and implementation of classification procedure. **Left:** Higher classification performance in MDD vs HC classification task can be achieved by implementing deep ML models, such as DenseNet, in comparison to a shallow ML model, for example, SVM. Furthermore, the analysis of integrated morphometric features can provide a more detailed description of cortical organization than separated features, leading to better differentiability of MDD from HC. The application of ComBat may improve the generalizability of results as site-related differences are removed. **Right:** Cortical sulcal depth, curvature, and thickness are first projected into the 2D grid and then transformed into 2D images using OMT projection. We split the data into 10 CV folds according to age and sex (Splitting by Age/Sex) and according to the site belonging (Splitting by Site). After the residualization step, where the age and sex effect are regressed out linearly, we train and test SVM and DenseNet on the diagnosis classification.

Each site acquired structural T1-weighted MRI scans of participants and preprocessed them according to ENIGMA Consortium protocol (http://enigma.ini.usc.edu/protocols/imaging-protocols/). This pipeline includes the segmentation of T1-weighted MRI volumes, tessellation, topology correction, and spherical inflation of the white matter surface. Detailed information on the acquisition protocols and scanner model in each cohort can be found in Supplementary Table 2. Cortical meshes were generated during FreeSurfer preprocessing in every site. Cerebral cortex meshes were then extracted from the FreeSurfer unsmoothed fsaverage6 template, effectively removing intracranial volume (ICV) differences (Supplementary Fig. 1) and yielding 37,747 and 37,766 vertices for the left and right hemispheres, respectively. The preprocessing pipeline applied in this study is consistent across all subjects, regardless of age, as the core procedures do not differ fundamentally between adolescents and adults. We analyzed vertex-wise features, such as sulcal depth, curvature, and thickness, both as integrated features and separately (Fig. 1).

Considering the absence of well-established pre-trained on cortical meshes CNN models, we projected 3D cortical surfaces into 2D images and applied DenseNet, which was pre-trained on natural images. There are few studies applying different projection methods such as latitude/longitude project and area-preserving maps [e.g., 26, 41]. Of note, the latitude/longitude method, in which cortical mesh is first re-sampled to the sphere and consequently mapped to the 2D grid, creates strong area distortions in the edges and near the medial wall close to subcortical regions [41]. Both methods may (differentially) influence subsequent classification performances, but to the best of our knowledge, no studies to date have directly compared this in one study using the same samples. Thus, we applied both 2D projection methods to the cortical meshes, resulting in 224 × 224 pixels images for each hemisphere. The images were normalized to present mean of 0 and standard deviation of 1.

### Data splitting

To assess potential biases in the model’s decision-making, we performed 10-fold cross-validation (CV) by splitting the data according to (1) demographic covariates, in which age and sex distribution were balanced and subjects from each site are equally distributed across all CV folds (*Splitting by Age/Sex*), and (2) site affiliation, where each site was contained only in one CV fold (*Splitting by Site*). In both strategies, 9 CV folds were used for training, while one remaining CV fold was used as a test set. This procedure was repeated iteratively until every CV fold was used as a test set. In the Splitting by Age/Sex strategy, effect of demographic factors on the classification performance is reduced, as the model is trained and tested on the same demographics. Nevertheless, the site-related differences may bias the decision-making of the classification models [20], which is directly addressed in Splitting by Site. This strategy demonstrates how well the model trained on one set of sites can be applied to the data from unseen sites. As the number of sites exceeds the number of folds, we distributed the sites across the folds to balance the number of subjects in every fold as close as possible by iteratively distributing the largest sites across all 10 folds. Smallest folds were added subsequently to further even the number of subjects in every fold. Overall, the difference in the classification results between these two splitting strategies may indicate the existence of the site effect. More detailed description of both splitting strategies can be found elsewhere [20].

### MDD vs HC classification

After the data-splitting step, the primary analysis was carried out. Firstly, we residualized all features normatively, removing linear age and sex dependencies. To avoid data leakage, age and sex regressors were estimated on the healthy subjects from the training set (9 CV folds) and then applied to the training and test set (1 CV fold) for patients and HC. Next, the classification algorithms were trained on the training folds, and classification performance was estimated on the test fold. As demonstrated by Dinga and colleagues, accuracy alone should be avoided as it does not account for class frequencies [47]. Thus, the algorithms were evaluated according to categorical measures, including balanced accuracy, sensitivity, specificity, and rank-based measure – AUC, allowing for a broad overview of performance. For model-level assessment [48], we performed the classification using all features combined and then using features separately to assess the final classification performance. We evaluated the classification performance of a robust shallow model - SVM with linear kernel, and DL model - DenseNet pre-trained on natural images from ImageNet dataset [49], which has been shown to be a robust convolutional neural network for image classification in both natural images and neuroimaging contexts [26, 43]. When DenseNet was trained on a single data domain, left and right hemisphere images were propagated through corresponding left and right DenseNets, the fully connected layers of which were concatenated. The resulting feature vectors were then fed to the output layer. For the whole-brain all-features analysis, we combined the features extracted from every feature and hemisphere, concatenate them, and fed them to the output layer. For SVM, all considered images were flattened and then concatenated into a single array. In this study, we intentionally chose not to apply dimensionality reduction techniques (e.g., PCA or feature selection) prior to model training. This decision was driven by the goal of preserving the full anatomical interpretability of vertex-wise cortical features and directly evaluating the classification potential of the complete morphometric representation. To mitigate the risk of overfitting in this high-dimensional setting, we implemented nested 10-fold cross-validation for robust performance estimation and hyperparameter tuning. Specifically, for the SVM, nine values of the regularization parameter (C) were explored, resulting in 90 model evaluations across outer folds. For DenseNet, the grid search spanned 54 unique hyperparameter combinations, yielding 540 model evaluations (hyperparameters in Supplementary Table 3). The concept and implementation of analysis are illustrated in Fig. 1. To mitigate site-related differences, which may potentially bias the classification results, we additionally performed the analysis by harmonizing all features via ComBat. Variance explained by age and sex was preserved during this harmonization step. Next, we residualized features normatively, as described above, and trained/tested the models. Application of ComBat differed for both splitting strategies. In short, ComBat parameters estimated on the training set were applied to the test set directly for the Age/Sex splitting strategy. In splitting by Site, ComBat was applied twice: first, we used ComBat to harmonize the training sites; second, we applied ComBat to adjust the test sites to the harmonized training sites, i.e., using the training sites as the reference batch [50]. A more detailed description of the ComBat application can be found in our previous work [20].

### Auxiliary analysis in projection methods

To explore and evaluate the potential impact of 2D projection methods on the classification performance, we compared both methods in their ability to classify healthy males from healthy females in 3 of the largest cohorts separately. The single-site classification was estimated via 10-fold CV on 411, 723, and 397 subjects, respectively. As usual, 9 CV folds were used for training, while one remaining CV fold was used as a test set. This procedure was repeated iteratively until every CV fold is used as a test set. To provide an initial perspective on the potential classification advantages of the pre-trained DenseNet, we presented the balanced accuracies obtained by two classifiers: an SVM with a linear kernel and the DenseNet [43]. Furthermore, using the hyperparameters found via the sex classification task (Supplementary Table 3), we presented the classification performance of both models.

## Results

### Participants and data splitting

We detected substantial differences in age (78% of pairwise comparisons between cohorts were significant, t-test, *p* < 0.05) and sex (47%, t-test, *p* < 0.05) across cohorts. The demographic and clinical profile is presented in Table 1. As expected, Splitting by Age/Sex resulted in more balanced CV folds with respect to number of subjects, age and sex distributions, while folds created by Splitting by Site were more uneven on these characteristics (Table 2).

**Table 1:** Participating sites.

Cohort	N HC	N MDD	Age HC (mean ± SD)	Age MDD (mean ± SD)	N females in HC (%)	N females in MDD (%)	Ethnicity White/Black/Asian/Other/NA HC	Ethnicity White/Black/Asian/Other/NA MDD
AFFDIS	44	33	39.98 ± 14.63	39.58 ± 15.42	20 (45.5%)	14 (42.4%)	NA	NA
Barcelona-StPau	32	62	46.03 ± 8.13	46.98 ± 7.75	23 (71.9%)	49 (79%)	16/0/0/0/16	62/0/0/0/0
CARDIFF	0	39	NA	46.67 ± 12.02	NA	26 (66.7%)	NA	NA
CSAN	49	60	33.2 ± 12.19	35.92 ± 13.49	34 (69.4%)	40 (66.7%)	NA	NA
Calgary	52	56	15.81 ± 5.08	18.15 ± 2.53	29 (55.8%)	32 (57.1%)	22/0/5/4/7	23/1/0/1/19
DCHS	70	22	31.46 ± 6.88	28.77 ± 6.34	70 (100%)	22 (100%)	0/66/0/0/4	0/21/0/0/1
FIDMAG	34	36	45.94 ± 11.67	48.61 ± 12.94	22 (64.7%)	22 (61.1%)	34/0/0/0/0	36/0/0/0/0
FOR2107Marburg	411	327	34.76 ± 12.78	35.6 ± 12.82	257 (62.5%)	208 (63.6%)	411/0/0/0/0	327/0/0/0/0
FOR2107Munster	221	174	28.34 ± 10.32	35.87 ± 12.92	140 (63.3%)	109 (62.6%)	221/0/0/0/0	174/0/0/0/0
Hiroshima	169	150	39.88 ± 12.39	44.25 ± 11.99	104 (61.5%)	71 (47.3%)	0/0/169/0/0	0/0/150/0/0
Houston	186	108	26.75 ± 15.97	32.31 ± 16.46	104 (55.6%)	66 (61.1%)	76/11/5/93/2	63/7/0/37/1
MODECT	0	27	NA	74.11 ± 9.81	NA	18 (66.7%)	NA	0/0/0/0/27
MOODS	32	64	35.12 ± 12.9	34.25 ± 12.45	21 (65.6%)	44 (68.8%)	25/1/2/0/0	45/11/4/0/0
Melbourne	9	22	20.67 ± 3.54	19.64 ± 3.11	3 (33.3%)	17 (77.3%)	0/0/0/0/9	2/0/0/0/20
Minnesota	40	70	15.68 ± 2	15.36 ± 1.84	26 (65%)	53 (75.7%)	25/1/3/11/0	49/7/1/13/0
Moraldilemma	46	24	18.5 ± 1.77	19.42 ± 2.19	46 (100%)	24 (100%)	20/2/14/2/8	18/0/4/2/0
Munster	723	282	35.36 ± 12.14	37.68 ± 12.03	413 (57.1%)	164 (58.2%)	723/0/0/0/0	282/0/0/0/0
NESDA	65	154	40.29 ± 9.74	37.19 ± 10.49	42 (64.6%)	103 (66.9%)	63/0/0/0/2	139/0/0/0/15
QTIM	286	100	21.99 ± 3.37	22.09 ± 3.17	184 (64.3%)	74 (74%)	286/0/0/0/0	100/0/0/0/0
UCSF	90	77	15.29 ± 1.29	15.61 ± 1.37	43 (47.8%)	51 (66.2%)	26/2/7/54/1	15/6/2/54/0
SanRaffaele	0	128	NA	49.84 ± 10.97	NA	84 (65.6%)	NA	128/0/0/0/0
SHIP_START	443	136	55.44 ± 12.82	53.59 ± 11.72	198 (44.7%)	96 (70.6%)	443/0/0/0/0	136/0/0/0/0
SHIP_TREND	937	312	50.64 ± 14.28	49.20 ± 12.15	409 (43.6%)	203 (65.1%)	937/0/0/0/0	312/0/0/0/0
Sexpect	20	20	33.75 ± 7.2	38.25 ± 11.63	3 (15%)	8 (40%)	NA	NA
Singapore	17	23	38.53 ± 4.64	39.3 ± 8.34	9 (52.9%)	10 (43.5%)	0/0/17/0/0	0/0/23/0/0
Socat_dep	99	79	36.55 ± 13.65	39.66 ± 12.89	89 (89.9%)	71 (89.9%)	99/0/0/0/0	79/0/0/0/0
StanfFAA	18	14	30.44 ± 10.25	35.63 ± 8.44	18 (100%)	14 (100%)	6/2/1/3/6	8/2/2/0/2
StanfT1wAggr	59	56	37.24 ± 10.52	37.11 ± 10.18	36 (61%)	33 (58.9%)	NA	NA
TAD	0	39	NA	16.03 ± 1.15	NA	29 (74.4%)	NA	24/2/7/5/1
TIGER	11	49	15.18 ± 1.08	15.73 ± 1.4	5 (45.5%)	33 (67.3%)	NA	NA
Jena (TiPs)	76	28	47.7 ± 16.14	43.36 ± 12.04	35 (46.1%)	14 (50%)	NA	NA

Cohort	Antidepressant free / users MDD	First / recurrent episode MDD	Remitted / acute episode MDD	BDI total score MDD (mean ± SD)	HDRS total score MDD (mean ± SD)	Age of onset MDD (mean ± SD)
AFFDIS	1/76	NA	0/77	19.22 ± 14.07	9.88 ± 7.06	NA
Barcelona-StPau	4/58	22/40	23/39	NA	13.66 ± 8.18	33.16 ± 11.43
CARDIFF	0/39	0/34	0/39	36.16 ± 9.33	19.58 ± 4.76	28.94 ± 14
CSAN	31/29	14/46	0/60	NA	NA	NA
Calgary	38/18	19/37	0/56	26.67 ± 11.53	19.16 ± 6.66	14.33 ± 3.2
DCHS	NA	NA	NA	NA	NA	NA
FIDMAG	4/31	11/23	1/35	NA	24.69 ± 5.78	37.06 ± 13.57
FOR2107Marburg	124/203	93/200	76/251	18.67 ± 10.82	8.06 ± 6.42	27.38 ± 13.42
FOR2107Munster	72/102	63/108	59/115	16.85 ± 11.72	9.72 ± 7.35	24.75 ± 11.15
Hiroshima	10/138	74/74	0/150	29.79 ± 9.39	18.67 ± 5.58	38.16 ± 13.25
Houston	105/1	21/43	39/37	16.5 ± 15.12	9.87 ± 7.93	21.57 ± 10.7
MODECT	19/8	0/22	0/27	NA	NA	NA
MOODS	64/0	32/32	0/64	NA	26.56 ± 5.37	28.25 ± 11.54
Melbourne	19/3	7/15	0/22	NA	NA	15.71 ± 3.85
Minnesota	52/16	16/22	6/0	25.85 ± 12.12	NA	12.39 ± 2.39
Moraldilemma	24/0	8/16	0/24	NA	NA	NA
Munster	27/231	64/216	23/258	25.63 ± 10.18	18.96 ± 4.3	29.35 ± 11.78
NESDA	98/56	67/87	0/154	NA	NA	24.17 ± 10.96
QTIM	70/30	NA	NA	NA	NA	18.42 ± 3.4
UCSF	77/0	32/36	7/61	26.68 ± 11.78	NA	13.25 ± 2.24
SanRaffaele	7/120	12/116	17/111	14.88 ± 8.05	20.26 ± 6.76	35.61 ± 12.32
SHIP_START	113/23	77/59	NA	11.56 ± 10.30	NA	38.01 ± 13.05
SHIP_TREND	258/54	113/199	NA	12.45 ± 8.11	NA	36.19 ± 14.27
Sexpect	0/20	4/16	0/20	20.86 ± 12.4	12.9 ± 5.11	30.87 ± 11.24
Singapore	5/18	8/15	NA	NA	6.3 ± 6.23	32.73 ± 9.51
Socat_dep	41/38	19/60	33/46	24.01 ± 12.14	13.29 ± 7.55	31.63 ± 16.67
StanfFAA	11/3	0/14	0/14	28.29 ± 9.98	18.86 ± 4.22	16.29 ± 6.83
StanfT1wAggr	27/20	6/48	0/56	25.76 ± 9.99	14.38 ± 5.84	19.52 ± 9.24
TAD	24/15	24/9	0/39	NA	NA	12 ± 2.52
TIGER	29/20	29/20	0/49	NA	NA	12.12 ± 2.49
Jena	10/18	5/23	NA	21.26 ± 11.71	NA	NA

The demographic and clinical information of participants across sites is presented.

*BDI* beck depression inventory, *HC* healthy controls, *HDRS* hamilton depression rating scale, *MDD* major depressive disorder, *SD* standard deviation, *N* number of participants.

**Table 2:** Data splitting strategies.

Splitting By Age/Sex				Splitting by Site			
Fold	Number of subjects	Mean age (SD)	Number of Females (%)	Fold	Number of subjects	Mean age (SD)	Number of Females (%)
0	708	38.34 (16.41)	434 (61)	0	1249	50.28 (13.78)	612 (49)
1	685	38.41 (16.51)	395 (58)	1	1005	36.01 (12.14)	577 (57)
2	692	38.59 (16.25)	441 (64)	2	738	36.30 (13.39)	465 (63)
3	709	37.99 (16.07)	428 (60)	3	579	55.00 (12.57)	294 (51)
4	704	38.74 (15.93)	417 (59)	4	563	33.06 (15.73)	374 (66)
5	708	38.90 (16.28)	415 (58)	5	596	26.42 (11.25)	370 (62)
6	693	38.09 (16.27)	423 (61)	6	559	36.89 (13.71)	372 (67)
7	716	38.3 (16.35)	431 (60)	7	589	35.71 (16.52)	356 (60)
8	689	38.55 (16.12)	396 (57)	8	546	28.70 (13.59)	359 (66)
9	708	38.14 (16.57)	406 (57)	9	588	33.99 (16.12)	407 (69)

Differences manifested in age/sex distribution and number of subjects between corresponding folds per splitting strategy.

### MDD vs HC classification

**Fig. 2:**
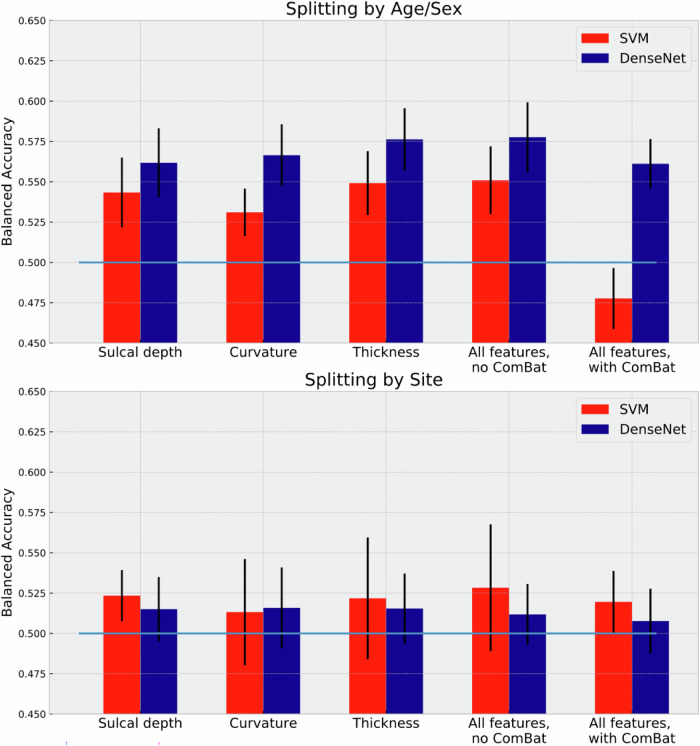
MDD vs HC classification performance of SVM and DenseNet applied to vertex-wise cortical features. Balanced accuracy for both classification models when trained on all features integrated with and without ComBat harmonization for both splitting strategies and when trained on single features. Error bars represent standard deviation.

First, we compared the performance of SVM and DenseNet for different splitting strategies (Fig. 2). In Splitting by Age/Sex, SVM achieved 0.551 ± 0.021 in balanced accuracy, while DenseNet yielded 0.578 ± 0.022. In Splitting by Site, both SVM and DenseNet models performed worse, yielding 0.528 ± 0.039 and 0.512 ± 0.019, respectively. The minor difference in classification performances for different splitting strategies indicated a potential site effect, which we addressed by applying ComBat. In Splitting by Age/Sex, the balanced accuracy of SVM with ComBat dropped to 0.478 ± 0.019, while the performance of DenseNet did not change and yielded 0.561 ± 0.015. In splitting by Site with ComBat, the performance of both models was similar and close to random chance, balanced accuracy yielded 0.520 ± 0.019 and 0.508 ± 0.020 for SVM and DenseNet respectively. Thus, we did not observe an improvement of models’ performances after data harmonization by ComBat. A full panel of results, including all classification metrics, can be found in Supplementary Table 4.

Next, we explored if any of the considered feature modalities yields greater classification performance (Fig. 2). In Splitting by Age/Sex, all data modalities yielded similar range of accuracies: thickness (SVM: 0.549 ± 0.020; DenseNet: 0.576 ± 0.019) compared to sulcal depth (SVM: 0.543 ± 0.022; DenseNet: 0.562 ± 0.019), and curvature (SVM: 0.531 ± 0.015; DenseNet: 0.567 ± 0.019), observed for both classification models. In Splitting by Site, sulcal depth (SVM: 0.523 ± 0.016; DenseNet: 0.515 ± 0.020), curvature (SVM: 0.513 ± 0.033; DenseNet: 0.516 ± 0.025) and thickness (SVM: 0.522 ± 0.038; DenseNet: 0.515 ± 0.022) also exhibited similar range of classification accuracies. Both models performed similarly for all feature types. These results demonstrate that integration of shape modalities with cortical thickness did not benefit the classification models. Results from the exploratory analyses for each hemisphere and for each feature modality per hemisphere showed no improvements in performance of the models (Supplementary Table 5, Supplementary Fig. 3). In addition, we applied the main demographic and clinical stratifications used in the ENIGMA-MDD working group to assess post-hoc whether groups that are more homogeneous would achieve better classification metrics (Supplementary Table 6).

### Auxiliary sex prediction task

As an initial step, we also conducted a sex classification to explore which projection method (latitude/longitude, OMT) yields higher classification performance for both SVM and DenseNet (Supplementary Fig. 2). There was no clear difference between projection methods; however, we observed a consistently higher classification performance of DenseNet compared to SVM for all types of features and hemispheres. Considering previous success of OMT projection as a projection method applied on cortical surface and its property to preserve distances between vertices [26], we conducted our main analysis with OMT projection.

## Discussion

In this work, we evaluated the diagnostic classification performance of DenseNet and SVM models, trained on cortical maps projected via OMT, including sulcal depth, curvature, and thickness, from a multi-site global dataset. Our analysis included 7012 participants from 31 sites worldwide, allowing for a comprehensive and realistic overview of classification performances. Both models were evaluated in parallel using two different CV splitting strategies. In Splitting by Age/Sex, we obtained CV folds with comparable demographics; thus, the performance of the models should not be affected by these demographic variables. In Splitting by Site, sites were distributed across folds. Therefore, models were trained and tested on different sets of sites. This strategy is closer to application of diagnostic classification models in clinical practice, and allowed for realistic estimation of classification performance on unseen sites. Overall, the classification performances of both models were similar: In Splitting by Age/Sex, DenseNet achieved 58 vs 55% for SVM; in Splitting by Site, the difference was even more negligible, DenseNet achieved 51 vs 52% for SVM. Both models performed better in Splitting by Age/Sex, implying the presence of a confounding site effect, most likely arising from differences in scanner vendors or image acquisition parameters. In this sense, ComBat approximated the classification results of the two splitting strategies, but did not improve the accuracy of the models. Ultimately, the classification performances of both models for all integrated morphometric features, both in Splitting by Age/Sex and in Splitting by Site, revealed similar classification levels of single-features.

### Cortical morphological maps as diagnostic biomarkers for MDD

To the best of our knowledge, this is the first study to combine cortical thickness, sulcal depth, and curvature features in order to classify MDD vs HC. Furthermore, previous ML studies with large samples only incorporated low-resolution atlas-based thickness characteristics. In our approach, we analyzed vertex-wise information, providing a richer and more detailed description of brain characteristics than atlas-derived regional measures. Even so, the integration of complementary cortical characteristics did not lead to higher classification performances compared to the accuracies obtained from the single cortical features, regardless of the data splitting strategy and the classification model. In Splitting by Site, no feature yielded an accuracy substantially higher than random chance accuracy, indicating the failure of both models to capture MDD-specific alterations. Furthermore, the analysis of finer-grained cortical maps, even for thickness alone, did not result in higher classification performance, compared to ML performance levels observed in our previous study [20]. Thus, the assumption that higher resolution would lead to greater classification performance did not hold in this study, as all results were close to the chance level, in line with previous attempts in classifying MDD [14, 15, 20]. Furthermore, stratification of the sample according to demographic (sex) and clinical characteristics (age of onset, antidepressant use, and number of depressive episodes) did not yield better differentiability between HC and MDD, in line with our previous study [20]. This new evidence suggests the absence of prominent gray matter alterations that alone may serve as diagnostic tool in patients with MDD. Combining features such as sulcal depth, curvature, and thickness in vertex-wise, unfolded cortical maps, and including them within a deep learning framework, is highly original. It advances prior work [24, 25, 27–29, 31] by integrating these complementary morphometric dimensions in a way few studies have, potentially clarifying whether these combined metrics can yield robust, clinically actionable biomarkers.

Although we combined complementary characteristics in the analysis, the interaction between thickness and shape was not addressed here. According to recent evidence, local cortical shape may correlate with thickness [51]. So, combined thickness-shape patterns should be further explored for the classification of MDD. Furthermore, reduced myelination was associated with MDD [52–54], which could lead to structural reorganization of cortical features, making it a potential classification aspect to be investigated. In addition, subcortical morphological characteristics may improve the classification by taking into account structural modifications in cortico-subcortical loops associated with MDD [8].

Integration of morphological characteristics with cytoarchitectonic and functional information may allow better contextualization of MDD-related alterations, as demonstrated in transdiagnostic study [55], with the potential to achieve higher classification performance [56, 57]. Brain topology can be described via the connectome - a whole-brain connectivity architecture of the brain. As nodes of brain connectome exhibited elevated susceptibility to brain disorders [58], graph analytical approaches could also lead to stronger differentiability between MDD and HC. Moreover, subject-specific parcellation schemes could be applied to compute structural and functional connectomes [59], and further analyzed by suitable sophisticated classification models taking into account the neural architecture e.g., with graph neural network [60].

### Data splitting and site effect

Several multi-site psychiatric neuroimaging studies directly demonstrated how different splitting strategies might introduce unwanted biases in inflated classification performances [20, 36, 61]. In Splitting by Age/Sex, trained models are unbiased regarding demographic factors; while in Splitting by Site the site affiliation is controlled, therefore we addressed the generalizability of the models applied to unseen sites. Similar to the results from our previous study [20], the classification performance of both SVM and DenseNet was higher in Splitting by Age/Sex, up to 58%, compared to Splitting by Site, close to random chance. This discrepancy indicates the existence of hidden site-related biases influencing classification performance. As this nuisance-based phenomenon appears in multi-site mega-analyses [36, 62] for its better comprehension, we strongly encourage the application of different splitting strategies in future multi-site ML studies.

The low accuracy of both models in Splitting by Site strategy is either due to the presence of a strong site-effect, hindering the ability of the models to capture diagnosis-related differences, or due to the general inability of both models to find meaningful alterations associated with MDD. Therefore, we addressed site-effect via ComBat. Thus, the possibility remains that subject-level prediction based on cortical features is unfeasible. As Combat has never been applied to vertex-wise cortical projections, we visually inspected its effect on a single pixel for every feature type (Supplementary Fig. 4). The application of ComBat resulted in more homogenous value distribution across cohorts, in line with previous studies analyzing the effects on atlas-based features [17, 20]. Nevertheless, this harmonization step did not lead to improvement in accuracies. While demographic covariates were preserved, ComBat may over-correct the data [63], causing a part of MDD-related associations to be removed along with the site-effect. Against this, more careful consideration of the site-effect is required in the future studies.

In Splitting by Age/Sex, the balanced accuracy of both models dropped (SVM: 55–48%; DenseNet: 58–56%) when ComBat was applied. The decrease of model’s performances near the levels in Splitting by Site indicates that initial higher classifications are most likely driven by site-related biases. To further validate this assumption, we performed the classification with balanced ratio between HC and MDD in every site in Splitting by Age/Sex, which resulted in close to random chance accuracies in DenseNet and SVM. Noticeably, DenseNet was less affected by the application of ComBat in the original analysis, reflecting potential non-linear site-related differences that remained in the dataset after harmonization, which is in line with previous findings [64]. Therefore, we recommend ComBat only be applied when combining more linear models, such as SVM, while more sophisticated models alone should directly incorporate site information as an additional input.

### SVM vs DenseNet

Previous ML mega-analyses based on structural MDD vs HC classifications considered only shallow linear and non-linear ML models, such as SVM, penalized logistic regression and decision tree [14, 15, 20]. In this study, we extended the diagnostic classification approach by comparing the performance of shallow linear model - SVM with a linear kernel to a highly non-linear deep DenseNet classifier applied to vertex-wise cortical information. The explorative results of sex classification applied to HC revealed higher classification performance of the DenseNet compared to the SVM (Supplementary Fig. 2) for all data modalities. The higher accuracy suggests that DenseNet was able to capture non-linear sex dependencies that were present in the cortical maps. The superiority of DenseNet over SVM in the sex classification task was in line with previous study conducted on the same vertex-wise cortical maps [26]. Conversely, another large sample study revealed no advantage of using any deep architectures over simpler models in predicting demographic factors [37]; therefore, further tests in even bigger samples are required. Nevertheless, in this study both models exhibited a similar range of accuracies, close to random chance, for the main task of MDD versus HC classification. Therefore, the application of DenseNet did not yield the expected improvement for detecting combined (nor separated) structural cortical features that discriminate patients from controls.

Similar performance of the linear SVM and non-linear DenseNet model may be due to the absence of non-linear interactions between different cortical regions, significant for the MDD detection. Furthermore, the analyzed sample is highly heterogeneous in terms of demographic and clinical covariates, potentially interfering with the main task and lowering the classification performance. In this vein, there are several possible directions for improving DenseNet performance. First, the considered model was pre-trained only on natural images from ImageNet. The model could be subsequently pre-trained on cortical projections from an independent large sample using immediate task, for example predicting sex as it was performed in Gao’s study [26]. Furthermore, one could use more than one intermediate task to optimize the weights of the neural network, for example, predicting demographic or clinical covariates. This approach is broadly known as multi-task learning [65], the usefulness of which in the neuroimaging domain was already demonstrated [19, 35].

Secondly, the multi-task approach could be used to “unlearn” undesired biases. In our analysis, site-related differences were removed via ComBat. One could train the network to perform the main task while unlearning the scanner parameters, as was successfully demonstrated by Dinsdale and colleagues [66]. Furthermore, one could replace the residualization step in the same manner by making the network unlearn age and sex dependencies. In line with our previous analysis, we linearly regressed out age and sex dependencies from the cortical features using normative approach [20]. Considering the greater performance of the DenseNet model in predicting sex, we can speculate the presence of non-linear male-female differences in cortical morphology. Thus, unlearning age- and sex-related dependencies could improve classification performance.

### Further strengths and limitations

Here we were interested in using a pre-trained deep learning model—specifically, DenseNet—to see if it could effectively classify major depressive disorder (MDD) from healthy brains using finer-grained, unfolded cortical surface maps, and whether such information, when combined, could offer complementary classificatory value compared to previously examined features. This approach extends the methodology of our prior study [20], where we employed more conventional structural MRI-derived features such as cortical thickness, surface area, and subcortical volumes from whole-brain regions-of-interest (ROIs). Our current approach is original and methodologically relevant, particularly in light of increasing interest in surface-based neuroimaging analyses that go far beyond standard ROI measures. And by employing more detailed cortical maps from different sources—such as sulcal depth and curvature—and projecting them in unfolded 2D space, we sought to assess whether such refinements in cortical representation could provide additional or differentially informative patterns for diagnostic classification. While the classification performance did not surpass that of previous studies, this negative finding is itself valuable, helping to delineate the boundaries of what these finer-grained representations currently offer in this domain.

Although we did not apply dimensionality reduction in the present analysis, we acknowledge that this remains a promising avenue for future research. Prior work has employed PCA, spherical harmonic decomposition [67], surface eigenmodes [68], cortical gradients [69], and deep generative models such as variational autoencoders [70] to reduce feature dimensionality while preserving meaningful structure. The potential impact of these dimensionality reduction approaches on classification performance should be explored in dedicated follow-up studies.

A potential limitation of this study is the absence of modeling based on MDD subtypes. While studies have proposed various subtyping schemes to address the clinical and biological heterogeneity of MDD, there is a wide range of subtyping approaches and most were derived from small samples (e.g., [71]), with limited replication or independent validation. For this reason, we intentionally chose not to include a subtyping step. This decision avoids reliance on uncertain stratification and reflects a key strength of the approach: classification performance could have direct clinical applicability, independent of MDD subtype definitions. Nonetheless, we acknowledge that the presence of unmodeled heterogeneity within the MDD group may have contributed to the lack of discriminative performance observed. Another important limitation is the lack of detailed ethnic and genetic information across the full sample. Sociocultural and genetic diversity are known to influence both brain morphology and disease expression, and their absence may affect the generalizability of the findings. These remain open challenges for future research aiming to enhance the specificity and robustness of neuroimaging-based classifiers for MDD. In particular, large-scale studies incorporating diverse populations and robust subtyping frameworks may offer insights with broader international applicability.

## Conclusion

In this study, we tested if more advanced classification algorithms applied to high-resolution morphometric shape characteristics can improve MDD vs HC classification. Splitting the data according to demographic variables and according to site allowed a comprehensive analysis of model’s performances and biases. We detected site effects, which we addressed at least partially with the ComBat harmonization tool, but did not increase classification metrics. Both shallow and deep ML models exhibited low, close to chance accuracies. Most importantly, the integration of high-resolution cortical thickness and shape features from vertices did not lead to greater classification performance over previously analyzed atlas-based cortical features. According to our results, it seems unlikely that structural MRI alone will provide diagnostic biomarkers of MDD. Thus, further investigation is required into the classification performance applied to the fusion of other MRI modalities, including fMRI and DWI.

## Supplementary information

Supplemental Material

## Data Availability

The datasets generated and/or analyzed during the current study are not publicly available due to site restrictions but data may be available from the corresponding sites on reasonable request.
