# Supplementary material for: Classification of major depressive disorder using vertex-wise brain sulcal depth, curvature, and thickness with a deep and a shallow learning model
Source: Mol Psychiatry. 2025 Oct 3;31(3):1517–29. doi: 10.1038/s41380-025-03273-w (PMC12916314; doi:10.1038/s41380-025-03273-w)
Supplement: Supplementary file 1 — Supplemental Material [file 41380_2025_3273_MOESM1_ESM.docx]

**Supplementary Material for**

**Classification of Major Depressive Disorder Using Vertex-Wise Brain Sulcal Depth, Curvature, and Thickness with a Deep and a Shallow Learning Model**

**Supplementary Table 1:** ENIGMA-MDD cohort-specific diagnostic instruments for major depressive disorder (MDD) and exclusion criteria

| **Cohort** | **Diagnosis measurment** | **Sample characteristics/Inclusion criteria** | **Exclusion criteria** |
| --- | --- | --- | --- |
| **AFFDIS** | ICD-10/DSM-IV criteria | MDD subjects currently depressed and in day program or inpatient | All subjects exclusion criteria: current or history of neurological disorder or brain injury, current substance abuse or dependence (not including nicotine), pregnancy, MRI contraindications, inability to give consent. MDD specific: comorbid psychiatric diagnosis. Healthy control specific: current or history of psychiatric diagnosis. |
| **Pharmo (AMC)** | MINI Plus | 48 subjects with lifetime diagnosis of either MDD and/or AD and 14 healthy controls. Patients were startified depending on exposure to SSRIs: early (before age 23) or late (after age 23) exposure to SSRI's, or no exposure at all (UN). 15 subjects were diagnosed with only MDD, 3 with only AD and 22 with both MDD and AD (8 subjects did not receive a diagnosis due to incomplete M.I.N.I. Plus assessment). According to the M.I.N.I. Plus, none of the HC subjects were ever diagnosed with MDD or AD | Less than three week medication-free interval before scanning, current psychotropic medication use, a history of chronic or neurological disorder, family history of sudden heart failure or epileptic attacks, pregnancy (tested via urine sampling prior to the assessment), breast feeding, alcohol dependence and contra-indications for an MRI scan (e.g., ferromagnetic fragments). Participants agreed to abstain from smoking, caffeine and alcohol use for 24 hours prior to the assessments. |
| **Barcelona** | DSM-IV-TR acc. to CIDI-interview and HAMD | Outpatients with MDD diagnosis (DSM-IV-TR), with a first episode, recurrent MDD or chronic MDD (TRD) age 18-65 | The exclusion criteria for healthy participants were: lifetime psychiatric diagnoses, first-degree relatives with psychiatric diagnoses and clinically significant physical or neurological illnesses. Axis I comorbidity according to DSM-IV-TR criteria was an exclusion criteria for all participants. |
| **Cardiff** | Hamilton Depression Rating Scale (HDRS-17) | N= 40, MDD patients with a current moderate to severe depressive episode despite minimum three months of stable antidepressant treatment | Psychotic symptoms, current substance dependence, eating disorders, claustrophobia and other MRI contraindications, and ongoing non-pharmacological treatment. |
| **CSAN (Adf)** | MINI | Current MDD: Meets MINI criteria for depression; comorbid anxiety disorders are allowed; mood-congruent psychotic symptoms allowed. | Current MDD: a current DSM-5 diagnosis of substance use disorder, except nicotine; a psychotic disorder, except depression with mood-congruent psychotic features; new antidepressant medication during the month before study participation (two months for fluoxetine); change of the dose of psychotropic medications over the last month (antidepressant and antipsychotic medication) or the last two months (mood stabilizers and anticonvulsants). |
| **Calgary** | KSADS | First episode MDD and healthy controls (Dalhousie sample). Recurrent MDD and healthy controls, recruited via referral from clinicians in Calgary, Alberta and through advertisements in local clinics and at the University of Calgary (Calgary sample). | Dalhousie Sample: A history of neurological illness, medical illness, claustrophobia, >21 year of age, or the presence of a ferrous implant or pacemaker. University of Calgary: Left handed; history of seizures, epilepsy or other neurological or psychiatric diagnoses (specifically bipolar disorder, psychosis, pervasive developmental disorder, eating disorders, PTSD); pregnancy |
| **DCHS** | MINI | Women over the age of 18 years, who were between 20 and 28 weeks pregnant, who presented at either of the two recruitment clinics, and who had no intention of moving out of the area within the following year, and were able to give written consent | 1) loss of consciousness longer than 30 minutes, 2) inability to speak English, 3) current/lifetime alcohol and/or substance dependence or abuse, 4) psychopathology other than PTSD and/or MDD, 5) traumatic brain injury, 6) standard MRI exclusion criteria |
| **FIDMAG** | DSM-IV-TR criteria | MDD patients within a current depressive episode (HDRS >= 17, only 1 patient was in remission), right-handed, age 18-65 | Patients were excluded (i) if they were left-handed; (ii) if they were younger than 18 or older than 65 years; (iii) if they had a history of brain trauma or neurological disease; (iv) if they had shown alcohol/ substance abuse within 12 months prior to participation; and (v) if they had undergone electroconvulsive therapy in the previous 12 months. |
| **FOR2107Marburg** | SCID-1 | Participants recruited by means of public advertisement and from the inpatient services. Inclusion criteria: age 18-65 years; patients were diagnosed with major depressive disorder by SCID-Interview, currently depressed or remitted. | Exclusion criteria all: any MRI contraindications; any neurological abnormalities. Exclusion criteria controls: any current or former psychiatric disorder; Exclusion criteria patients: substance dependence or current benzodiazepine treatment (wash out of at least three half-lives before study participation)" |
| **FOR2107Munster** | SCID-1 | Participants recruited by means of public advertisement and from the inpatient services. Inclusion criteria: age 18-65 years; patients were diagnosed with major depressive disorder by SCID-Interview, currently depressed or remitted. | Exclusion criteria all: any MRI contraindications; any neurological abnormalities. Exclusion criteria controls: any current or former psychiatric disorder; Exclusion criteria patients: substance dependence or current benzodiazepine treatment (wash out of at least three half-lives before study participation)" |
| **Houston** | SCID interview | Outpatients | MDD subjects: age below 18; lifetime or current diagnosis of psychotic disorder, or bipolar I or II disorder; substance abuse/dependence in 6 months prior to study inclusion; current major medical problems. Control subjects: age below 18; current major medical problems; current psychiatric or neurologic disorder; history of psychiatric disorders in a first-degree relative; current major medical problems. Both groups: MRI contra-indications |
| **Hiroshima** | MINI | MDD Patients were recruited from local clinics, 20-80 years. Controls were recruited from local community by advertising in local papers. | MDD patients: comorbid psychiatric disorders other than MDD, Control subjects: any history of psychiatric disorder |
| **TiPs (Jena, Germany)** | SCID interview | Psychiatric inpatients and tinnitus patients with MDD or a disorder of the depressive spectrum (also adjustment disorders as pointed out in the data table); psychiatrically healthy controls were derived from community and tinnitus patients | MDD subjects: presence of axis-I disorders other than MDD or adjustment disorders. Control subjects: no Axis-I diagnosis, no medication use. Exclusion criteria for all subjects included history of neurological disease (e.g. tumour, head trauma, epilepsy) or untreated internal medical condtitions, intellectual and/or developmental disability. Only German native speakers were allowed to participate. |
| **MODECT** | MINI | Older adults, aged above 55, with severe depression admitted to be treated with ECT | Exclusion criteria were another major DSM-IV-TR diagnosis, such as schizophrenia, bipolar or schizoaffective disorder and a history of major neurological illness (including Parkinson’s disease, stroke and dementia). |
| **Melbourne** | SCID interview | Youth depression sample: 15-25 years of age. Recruited as part of 2 large RCTs (incl. YoDA-C - Davey et al., 2014; Trials) and scanned prior to treatment randomisation. 60 patients unmedicated (YoDA-C). | MDD subjects: lifetime or current SCID-I diagnosis of psychotic disorder, or bipolar I or II disorder. Control subjects: any SCID-I diagnosis or medication use. Both groups: Acute or unstable medical disorder; general MRI contraindications |
| **Minnesota** | Schedule for Affective Disorders and Schizophrenia for School-Age Children–Present and Lifetime Version and the Children’s Depression Rating Scale–Revised (CDRS-R). | Adolescents with MDD and HCs aged 12 to 19 years were recruited to participate through community postings and referrals from local mental health services. Adolescents with MDD were eligible if they had a primary diagnosis of MDD and had not received any psychotropic medication treatment for the past 2 months. Healthy adolescents were eligible if they had no current or past psychiatric diagnoses and were frequency matched to the MDD group on age and sex | Exclusion criteria for both groups included the presence of a neurologic or other chronic medical condition, mental retardation, pervasive developmental disorder, substance use disorder, bipolar disorder, or schizophrenia |
| **MOODS / DEP-ARREST CLIN** | MINI, DSM5 | Patients aged 18-65 years with a current MDE diagnosis (MINI interview(Sheehan et al., 1998) and a minimum depression score of 18 on the Hamilton Depression Rating Scale-17 items (HDRS) in the context of MDD, as well as free of antidepressant drug use at least one month before the study beginning, were included. HCs were included based on the absence of current or past mental disorders or somatic conditions, particularly nasal polyposis and chronic or acute sinusitis or rhinitis | Patients suffering from bipolar disorder, psychotic disorder, eating disorder, and addictions, according to the DSM-5 criteria, or from nasal polyposis, chronic or acute sinusitis, chronic or acute rhinitis or pregnancy or breastfeeding, were not included. HCs were included based on the absence of current or past mental disorders or somatic conditions, particularly nasal polyposis and chronic or acute sinusitis or rhinitis |
| **Moral Dilemma** | SCID interview | Youth depression sample: 15-25 years of age; recruited from outpatient service. Controls recruited from general community. | MDD subjects: lifetime or current SCID-I diagnosis of psychotic disorder, or bipolar I or II disorder; current antidepressant medication use. Control subjects: any SCID-I diagnosis or medication use. Both groups: Acute or unstable medical disorder; general MRI contraindications |
| **Munster** | SCID interview | Participants recruited by means of public advertisement and from the inpatient services. Inclusion criteria: age 16-65 years; patients were diagnosed with major depressive disorder by SCID-Interview | MDD subjects: presence of bipolar disorder, schizoaffective disorders and schizophrenia; substancerelated disorders or current benzodiazepine treatment (wash out of at least three half-lives before study participation), and former electroconvulsive therapy. Control subjects: any current or former psychiatric disorder. Both groups: any neurological abnormalities, MRI contra-indications |
| **NESDA** | CIDI interview | DSM-4 based diagnosis of MDD (6 month recency), using CIDI interview. 93 (60%) MDD patients have a comorbid ANX diagnosis. Age range 18-65 | N/A |
| **QTIM** | CIDI interview | Retrospective questionnaire about depression episodes combined with an MRI study. The best described MDD episode is defined as the worst one (according to individuals). We have up to 5 supplementary episodes (briefly) described. Sample composed of twins and relatives. Population-based sample | MDD subjects: presence of axis-I disorders other than MDD and anxiety disorders Control subjects: antidepressant use, psychiatric disorders All subjects: relatedness between subjects, left handedness, history of neurological or other severe medical illness, head injury or current or past diagnosis of substance abuse, use of cognition affecting medication and general MRI contraindications |
| **San Francisco UCSF** | KSADS (semi-structured interview based on DSM) for MDD, DISC/DPS for HCL | Outpatient/community-based sample with DSM diagnosis, mostly antidepressant-naive and approximately 60% of MDD have comorbid anxiety disorders | Exclusion criteria for all participants included: 1) use of pharmacotherapeutics for treating psychiatric conditions within the past 6 months, 2) misuse of drugs within two months prior to MRI scanning; 3) two or more alcoholic drinks per week within the previous month (as assessed by the Customary Drinking and Drug Use Record; CDDR) (Brown et al, 1998); 4) a full scale IQ score of less than 75 (as assessed by the Wechsler Abbreviated Scale of Intelligence; WASI) (Wechsler, 1999); 5) contraindications for MRI including ferromagnetic implants and claustrophobia; 6) pregnancy or the possibility of pregnancy; 7) left-handedness; 8) prepubertal status (as assessed as Tanner stages of 1 or 2) (Tanner, 1962); 9) inability to understand and comply with procedures; 10) neurological disorder (including meningitis, migraine, or HIV); 11) head trauma; 12) learning disability; 13) serious health problems; and 14) complicated or premature birth (i.e., birth before 33 weeks of gestation). The MDD group was subject to the additional exclusion criterion of a primary psychiatric diagnosis other than MDD. The HCL group was subject to the additional exclusion criteria of: 1) history of mood or psychotic disorders in a first- or second-degree relative (as assessed by the Family Interview for Genetics; FIGS) (Maxwell, 1992); and 2) current or lifetime DSM-IV-TR Axis I psychiatric disorder. |
| **SHIP_START-2** | M-CIDI interview | Population based longitudinal cohort study | MDD subjects: presence of axis-I disorders other than MDD, anxiety disorders, conversion, somatization and eating disorder. Control subjects: no lifetime diagnosis of depression, no antidepressiva, and severity index=0 All subjects: We removed subjects with medical conditions (e.g. a history of cerebral tumor, stroke, Parkinson’s diseases, multiple sclerosis, epilepsy, hydrocephalus, enlarged ventricles, pathological lesions) or due to technical reasons (e.g. severe movement artifacts or inhomogeneity of the magnetic field). |
| **SHIP_TREND-0** | M-CIDI interview | Population based longitudinal cohort study | MDD subjects: no special exclusion criteria Control subjects: no lifetime diagnosis of depression, no antidepressiva, and severity index=0 All subjects: We removed subjects with due to medical conditions (e.g. a history of cerebral tumor, stroke, Parkinson’s diseases, multiple sclerosis, epilepsy, hydrocephalus, enlarged ventricles, pathological lesions) or due to technical reasons (e.g. severe movement artifacts or inhomogeneity of the magnetic field). |
| **Singapore** | SCID interview | Inclusion: 1) DSM IV dx of MDD (Patients) 2) Age: 21-65 3) English speaking 4) Provision of informed written consent | Exclusion criteria 1) History of significant head injury 2)Neurological diseases such as epilepsy, cerebrovascular accident 3) Impaired thyroid function 4) Steroid use 5) DSM IV alcohol or substance use or dependence 6) Contraindications to MRI (e.g. pacemaker, orbital foreign body, recent surgery/procedure with metallic devices/implants deployed) using standard MRI Request Form from NNI 7)Pregnant women 8) Claustrophobia |
| **SoCAT** | SCID interview | Inclusion criteria: DSM IV dx for mdd patients Age: 18-65 right-handed currently depressed or remitted; Control subjects: any history of psychiatric disorder | Exclusion criteria 1) History of significant head injury 2)Neurological diseases such as epilepsy, cerebrovascular accident 3)Other diagnoses on Axis I disorders4) |
| **Stanford FAA** | SCID interview | Community-based DSM-diagnosed sample | MDD subjects: presence of axis-I disorders other than MDD, anxiety and eating disorders . Control subjects: control individuals did not meet diagnostic criteria for any current psychiatric. Both groups: alcohol / substance abuse or dependence within six months prior to MRI scanning, history of head trauma with loss of consciousness > 5 min, aneurysm, or any neurological or metabolic disorders that require ongoing medication or that may affect the central nervous system (including thyroid disease, diabetes, epilepsy or other seizures, or multiple sclerosis), MRI contraindications, or bad MRI data (e.g., extreme movement). |
| **Stanford T1w Aggregate** | SCID interview | Community-based DSM-diagnosed sample | MDD subjects: presence of axis-I disorders other than MDD, anxiety and eating disorders . Control subjects: control individuals did not meet diagnostic criteria for any current psychiatric. Both groups: alcohol / substance abuse or dependence within six months prior to MRI scanning, history of head trauma with loss of consciousness > 5 min, aneurysm, or any neurological or metabolic disorders that require ongoing medication or that may affect the central nervous system (including thyroid disease, diabetes, epilepsy or other seizures, or multiple sclerosis), MRI contraindications, or bad MRI data (e.g., extreme movement). |
| **TAD** |  |  |  |
| **TIGER** | KSADS | Community-based DSM-diagnosed sample | All subjects: Exclusion criteria were premenarchal status (for females), history of concussion within the past 6 weeks or history of any lifetime concussion with loss of consciousness, contraindications to MRI scanning (e.g. braces, metal implants, or claustrophobia), serious neurological or intellectual disorders that could interfere with the participant's ability to complete study components. MDD subjects: meeting lifetime or current DSM-IV criteria for any Bipolar Disorder, Psychosis, or Alcohol Dependence, or DSM-5 criteria for Moderate Substance Use Disorder with substance-specific threshold for withdrawal. CTL subjects: any current or past DSM-IV Axis I Disorder and first-degree relative with confirmed or suspected history of depression, mania, psychosis, or substance dependence. |

**Supplementary Table 2**: ENIGMA-MDD cohort-specific MRI acquisition parameters and image processing protocols

| **Cohort** | **Scanner type** | **Sequence T1** | **FreeSurfer version** | **Slice orientation** | **Operating system** |
| --- | --- | --- | --- | --- | --- |
| **AFFDIS** | 3T Siemens Magnetom TrioTim | 3D T1 (176 slices; TR = 2250 ms; TE = 3.26 ms; FOV 256; voxel size 1X1X1mm) | 5,3 | Sagittal | Linux CentOS |
| **Barcelona** | 3T Philips Achieva | 3D MPRAGE images (Whole-brain T1-weighted); TR=6.7ms, TE=3.2ms; 170 slices, voxel size 0.89X0.89X1.2 mm. Image dimensions 288X288X170; field of view: 256X256X204; slice thickness: 1.2 mm; with a sagittal slice orientation, T1 contrast enhancement, flip angle: 8º, grey matter as a reference tissue, ACQ matrix MXP = 256X240 and turbo-field echo shots (TFE) = 218. | 6 | Sagittal | Scientific Linux 5 |
| **Cardiff** | A 3 Tesla whole body MRI system (General Electric, Milwaukee, USA) with an 8-channel head coil was used at the Cardiff University Brain Research Imaging Centre (CUBRIC). | High-resolution anatomical scan (Fast Spoiled Gradient-Recalled-Echo [FSPGR] sequence): 178 slices, TE=3 ms, TR=7.9 ms, voxel size=1.0×1.0×1.0 mm3, FA=15°, FOV=256×256 | 5,3 |  | freesurfer-Linux-centos6_x86_64-stable-pub-v5.3.0 |
| **CSAN (Adf)** | 3T Siemens MAGNETOM PRISMA | Whole-head t1-weighted MPRAGE (TR = 2300 ms, TE = 2.34 ms, FOV 250 × 250 mm, voxel size = 0.9 × 0.868 × 0.868 mm, flip angle = 8°) | 7.2 | Sagittal | Ubuntu |
| **Calgary** | 1.5T Siemens Magnetom Vision. 3T GE Discovery MR750 | 1.5T: A sagittal scout series was acquired to test image quality. 3D fast low angle shot (FLASH) sequence was used to acquire data from 124 1.5 mm-thick contiguous coronal slices through the entire brain (echo time = 5ms, repetition time = 25ms, acquisition matrix = 256 x 256 pixels, field of view = 24 cm and flip angle = 40°). 3T: Anatomical imaging acquisition parameters: axial acquisition, repetition time (TR), 2200 milliseconds (ms); echo time (TE), 3.04 ms; TI, 766, 780; flip angle, 13 degrees; 208 partitions; 256 × 256 matrix; and field of view, 256. | 5,3 | Dalhousie sample, coronal; Calgary sample, axial | MacOs Sierra |
| **DCHS** | 3T Siemens Skyra | 3D multi-echo MPRAGE, voxel size 1 mm x 1mm x 1.5mm, TR = 2530 ms, TE = 1.69 x 3.55 x 5.41 x 7.27ms, FOV: 256x256mm, flip angle = 7° | 5.3 | Sagittal | Linux-centos6_x86_64 |
| **FOR2107 - Marbourg** | 3T Siemens Magnetom TiroTim syngo MR B17 | Sequence: 3D T1-weighted magnetization prepared rapid acquisition gradient echo (MPRAGE) - Sagittal Acquisition Direction, # of Slices 176, 0.5mm Slice Gap, 1.0x1.0x1.0 Voxel Size (mm3), TI 900 ms, TE 2.26 ms, TR 1900 ms, Flip Angle 9. | 5,3 | Sagittal | Red Hat Enterprise Linux Server release 5.11 (Tikanga) |
| **FOR2017 - Münster** | 3T Philips | 3D T1-weighted scan (170 slices; TR = 9ms; TE = 3.6ms; 256x231 matrix of 1×1×1 mm voxels) | 5,3 | Sagittal | Red Hat Enterprise Linux Server release 5.11 (Tikanga) |
| **FIDMAG** | 1.5T, GE Signa | 3D T1: matrix size = 512 × 512, 180 contiguous axial slices, voxel resolution = 0.47 × 0.47 × 1mm, no slice gap, TE = 3.93ms, TR = 2000ms and inversion time (TI) = 710ms, flip angle = 15 degrees | 6 | Axial | Linux-centos6_x86_64 |
| **Houston** | subjects in 20000s: 1.5 T Philips Medical Systems Gyroscan Intera; subjects in 30000s: 3T Siemens Allegra | Subjects in the 20000s: Fast field echo sequence- repetition time (TR) = 24 ms, echo time (TE) = 4.99 ms, flip angle = 40°, slice thickness = 1 mm, matrix size = 256 × 256 and 150 slices. Subjects in 30000s: MPRAGE- repetition time (TR) = 1750 ms, echo time (TE) = 4.39 ms, flip angle = 8°, slice thickness = 1 mm, matrix size = 208 × 256 and 160 slices. | 5,3 | Subjects in 20000s: Sagittal; Subjects in 30000s: Transverse | Fedora 19 |
| **Hiroshima** | 3T Siemens (Spectra, Verio.Dot), 3T GE (Signa HDxt) Site 1 = GE Signa HDxt 3.0T 2= GE Signa HDxt 3.0T 3 = SIEMENS MAGNETOM Spectra 3.0T 4 = SIEMENS MAGNETOM Verio.Dot 3.0T | T1 256x256x256 matrix of 1x1x1mm voxels (Siemens: ADNI MPRAGE (tfl), GRAPPA, 192 slices, GE: SPGR, 184 slices) *Detailed scanning parameter sheets are available for all 4 scanners on request. | 5,3 | Sagittal | Linux_Ubuntu_18.04 |
| **TiPs (Jena, Germany)** | 3T Siemens MAGNETOM Prisma_fit | MPRAGE sequence: TR 2300 ms, TE 3.03 ms, α 9°, 192 contiguous sagittal slices, in-plane field of view 256 mm, voxel resolution 1Å~1Å~1 mm; acquisition time 5:21 min | 5,3 | Sagittal | Linux |
| **MODECT** | 3T (General Electric Signa HDxt, Milwaukee, WI, USA) | T1-weigthed dataset was acquired (flip angle=12°, repetition time=7.84 milliseconds, echo time=3.02 milliseconds; matrix 256x256, voxel size 0.94x0.94x1 mm; 180 slices). | 5,3 | Coronal | Linux |
| **Melbourne** | 3T GE Signa Excite | 3D BRAVO sequence 140; TR=7900 ms; TE=3000 ms; flip angle=13º; FOV=256 mm; matrix=256 x 256 | 5,3 | Axial | Linux Debian x86 64 |
| **Minnesota** | 3.0 Tesla Tim Trio scanner; Siemens Corp | A 5-minute structural scan was acquired using a T1-weighted, high-resolution, magnetization-prepared gradient-echo sequence: repetition time, 2530 milliseconds; echo time, 3.65 milliseconds; inversion time, 1100 milliseconds; flip angle, 7°; field of view, 256 × 176 mm; voxel size, 1-mm isotropic; 224 slices; and generalized, autocalibrating, partially parallel acquisition acceleration factor, 2. | 5,3 | Coronal | Linux |
| **MOODS / DEP-ARREST CLIN** | 3T Philips Achieva | 3D T1-weighted image: TR=7, TE=3.5, FOV=352x352x180, Flip angle=8 degrees, number of slices : 180 slices, Slice gap 1 mm, voxel size: 0.8x0.8x1 | 6 | Transverse (Axial) | CentOS Linux 7 |
| **Moral Dilemma** | 3T GE Signa Excite | 3D BRAVO sequence: 140 contiguous slices; repetition time, 7900 ms; echo time, 3000 ms; flip angle, 13°; in a 25.6-cm field of view, with a 256 × 256 pixel matrix and a slice thickness of 1 mm (1 mm gap). | 5,3 | Axial | Linux Debian x86 64 |
| **Munster** | 3T Philips Gyroscan Intera | 3D fast gradient echo sequence (turbo field echo), repetition time = 7.4 milliseconds, echo time = 3.4 milliseconds, flip angle = 9°, two signal averages, inversion prepulse every 814.5 milliseconds, acquired over a field of view of 256 (feet -head [FH]) × 204 (anterior -posterior [AP]) × 160 (right -left [RL]) mm, phase encoding in AP and RL direction, reconstructed to cubic voxels of .5 mm × .5 mm × .5 mm | 5,3 | Sagittal | Red Hat Enterprise Linux Server release 5.11 (Tikanga) |
| **NESDA** | 3T Phillips Achieva/Intera | 3D gradient-echo T1-weighted sequence. TR=9 msec; TE=3.5 msec; flip angle 8º, FOV = 256 mm; matrix: 25x62x56; in plane voxel size = 1 mm × 1 mm x 1 mm; 170 slices. | 5 | Sagittal | SHARK HPC, Linux environment |
| **QTIM** | Bruker 4T Wholebody MRI | 3D T1 weighted sequence. TR=1500 msec; TE=3.35 msec; flip angle=8°, 256 or 240 (coronal or sagittal) slices, FOV=240 mm, matrix 256x256x256 (or 256x256x240) | 5,1 | Coronal, then sagittal following software upgrade. | Linux- centos4_x86_64- stable-pub-v5.1.0 |
| **San Francisco UCSF** | 3T GE Discovery MR750 | SPGR T1-weighted: TR=8.1 ms; TE=3.17 ms; TI=450 ms; flip angle=12°; 256x256 matrix; FOV=250x250 mm; 168 sagittal slices; slice thickness=1 mm; in-plane resolution=0.98x 0.98 mm | 5,3 | Sagittal | Linux-centos6_x86_64-stable-pub-v5.3.0. |
| **SHIP** **_START-2** | 1.5T Siemens Avanto | 3D T1-weighted (MP-RAGE/ axial plane); TR=1900 msec; TE=3.4 msec; Flip angle=15°; voxel size 1 mm x 1 mm x 1 mm | 5.3 (cortical), 5.1 (subcortical) | Axial | Centos6_x86_64 |
| **SHIP-** **_TREND-0** | 1.5T Siemens Avanto | 3D T1-weighted (MP-RAGE/ axial plane); TR=1900 msec; TE=3.4 msec; Flip angle=15°; voxel size 1 mm x 1 mm x 1 mm | 5.3 (cortical), 5.1 (subcortical) | Axial | Centos6_x86_64 |
| **Singapore** | Achieva 3T, Philips Medical Systems, Netherlands | Whole brain high resolution 3D MP-RAGE (magnetisation-prepared rapid acquisition with a gradient echo) volumetric scans (TR/TE/TI/flip angle 8.4/3.8/3000/8; matrix 256x204; FOV 240mm2) with axial orientation (reformatted to coronal) | 5,3 | Axial | Linux_Ubuntu12.04_6 4 |
| **SoCAT** | 3.0 T, Siemens Verio,Numaris/4,Syngo MR B17,Erlangen,Germany | 3D T1 weighted MP-Rage/axial plane; TR=1900 msec; TE=3.4 msec; Flip angle=15°; Voxel size 1 mm x 1 mm x 1 mm | 5,3 | Axial | Ubuntu 18.04 LTS |
| **Stanford FAA** | 3.0T GE Discovery MR750 | Whole-brain T1-weighted images were collected using a spoiled gradient echo (SPGR) pulse sequence (186 sagittal slices; resolution = 0.9 mm isotropic; flip angle = 12°; repetition time [TR] = 6,240 ms; echo time [TE] = 2.34 ms) | 5,3 | Sagittal | Linux-centos6_x86_64 |
| **Stanford T1w Aggregate** | 1.5T GE Signa Excite | Whole-brain T1-weighted images were collected using a spoiled gradient echo (SPGR) pulse sequence (116 sagittal slices; through-plane resolution = 1.5 mm; in-plane resolution = 0.86 x 0.86 mm; flip angle = 15 degrees; repetition time [TR] = 8.3-10.1 ms; echo time [TE] = 1.7-3.0; inversion time [TI] = 300 ms; matrix = 256 x 192). | 5,3 | Sagittal | Centos6_x86_64, Linux-based HPC |
| **TAD** |  |  |  |  |  |
| **TIGER** | 3T GE MR750 | TR/TE/TI=8.2/3.2/600 ms; flip angle=12°; 156 axial slices; FOV=25.6 cm; matrix=256 mm x 256 mm, isotropic voxel=1 mm, total scan time: 3:40 | 6 | Axial | Linux |

**Supplementary Table 3:** List of hyperparameters for trained algorithms used in the sex classification task. Optimal hyperparameters were identified via grid search. The range of values for the regularization parameter C was determined using a heuristic approach as outlined in Hsu et al. (2003).

| **Classification algorithm** | **Feature Selection** | **Hyperparameters** | **Nested CV** |
| --- | --- | --- | --- |
| **SVM Linear** | None | C = [${10}^{-4},{10}^{-3}, \ldots, {10}^{4}$] | 10 fold |
| **DenseNet** | None | Number of dense layers = [1,2,3]  Number of nodes in the dense layers = [10,100,200]  Adam optimizer: learning rate [.01,**.**001,.0001]  DropOut layer before dense layers (yes, no) | 10 fold |

**Supplementary Table 4:** Comparison of SVM and DenseNet classification performance on the full dataset using integrated whole-brain feature modalities. Performance metrics—including balanced accuracy, sensitivity, specificity, and AUC—are reported for each data splitting strategy, with and without ComBat harmonization.

|  | Splitting by Age/Sex | | Splitting by Site | |
| --- | --- | --- | --- | --- |
|  | No ComBat | With ComBat | No ComBat | With ComBat |
| SVM    Balanced Acc  Sensitivity  Specificity  AUC | 0.551 ± 0.021  0.477 ± 0.036  0.625 ± 0.030  0.566 ± 0.021 | 0.478 ± 0.019  0.420 ± 0.024  0.536 ± 0.021  0.490 ± 0.020 | 0.528 ± 0.039  0.490 ± 0.114  0.566 ± 0.124  0.536 ± 0.062 | 0.520 ± 0.019  0.465 ± 0.033  0.574 ± 0.049  0.520 ± 0.022 |
| DenseNet  Balanced Acc  Sensitivity  Specificity  AUC | 0.578 ± 0.022  0.452 ± 0.102  0.704 ± 0.104  0.606 ± 0.026 | 0.561 ± 0.015  0.401 ± 0.090  0.721 ± 0.092  0.595 ± 0.020 | 0.512 ± 0.019  0.428 ± 0.172  0.596 ± 0.217  0.549 ± 0.076 | 0.508 ± 0.020  0.466 ± 0.265  0.550 ± 0.241  0.544 ± 0.092 |

**Supplementary Table 5:** Major depressive disorder (MDD) vs. healthy control (HC) classification by hemisphere, assessing whether features from individual hemispheres resulted in higher classification accuracy.

|  | Splitting by Age/Sex | | Splitting by Site | |
| --- | --- | --- | --- | --- |
| Hemisphere | **Left** | **Right** | **Left** | **Right** |
| SVM | 0.546 ± 0.022 | 0.539 ± 0.017 | 0.514 ± 0.034 | 0.522 ± 0.036 |
| DenseNet | 0.569 ± 0.019 | 0.556 ± 0.024 | 0.513 ± 0.018 | 0.506 ± 0.017 |

**Supplementary Table 6:** Major depressive disorder (MDD) vs. healthy control (HC) classification stratified by key demographic and clinical characteristics, assessing post-hoc whether more homogeneous subgroups yielded improved classification accuracy.

|  |  | Splitting by Site | |
| --- | --- | --- | --- |
|  |  | **SVM** | **DenseNet** |
| Sex | Women (MDD n = 1,803; HC n = 2,383) | 0.516± 0.025 | 0.524± 0.018 |
|  | Men (MDD n = 950; HC n = 1,857) | 0.513± 0.032 | 0.517± 0.044 |
| Age of onset | Adolescent (MDD n = 1,096; HC n = 4,240) | 0.547± 0.059 | 0.530± 0.102 |
|  | Adults (MDD n = 1,302; HC n = 4,231) | 0.506± 0.070 | 0.579± 0.187 |
| Number of episodes | Recurrent (MDD n = 1,624; HC n = 4,240) | 0.543± 0.040 | 0.515± 0.043 |
|  | Single (MDD n = 900; HC n = 4,240) | 0.492± 0.024 | 0.500± 0.020 |
| AD use | No (MDD n = 1,224; HC n = 4,240) | 0.503± 0.026 | 0.490± 0.037 |
|  | Yes (MDD n = 1,313; HC n = 4,240) | 0.552± 0.049 | 0.544± 0.054 |


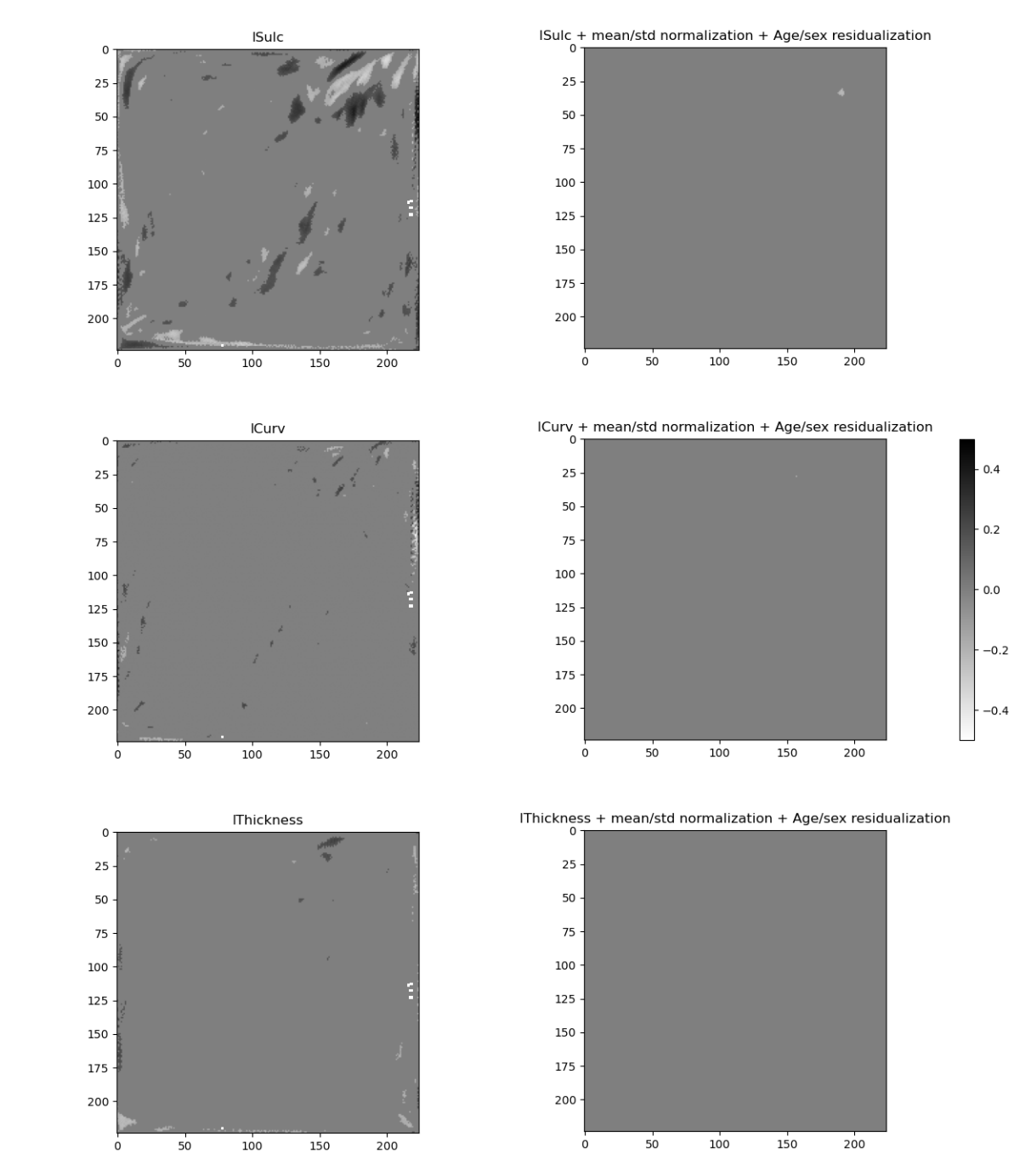


**Supplementary Figure 1:** Intracranial volume (ICV) dependence in cortical feature maps. To assess whether ICV is encoded in cortical features, we correlated ICV values with pixel intensities from SHIP_TREND_0 healthy controls (left panel). After standardizing the features (mean = 0, SD = 1) and regressing out age and sex, the ICV effect was effectively removed (right panel). The colormap indicates the direction of significant correlations.


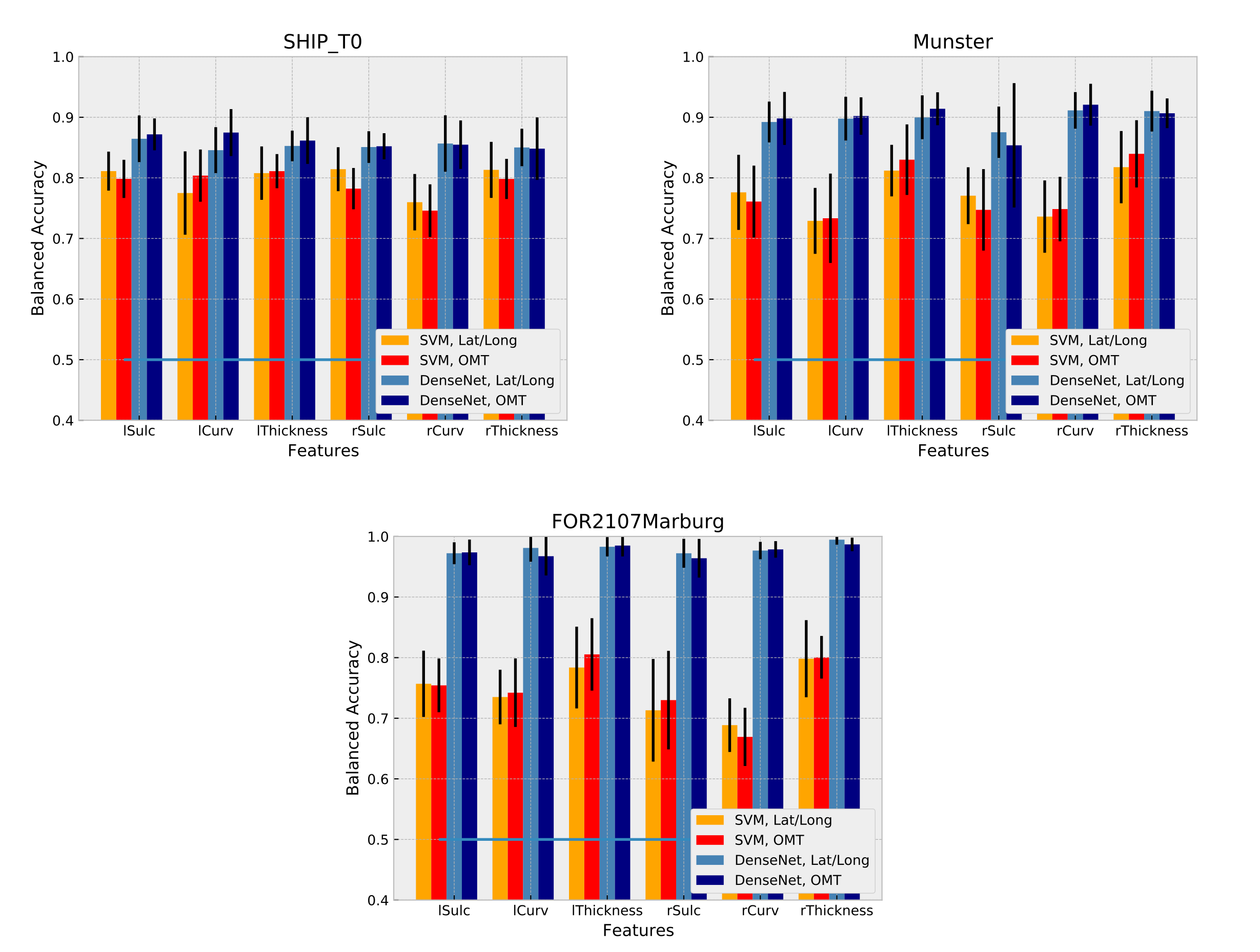


**Supplementary Figure 2:** Sex classification performance across major cohorts. Balanced accuracy of SVM and DenseNet classifiers was estimated separately for each feature type in the three largest cohorts: SHIP_TREND-0 (top left), Münster (top right), and FOR2107-Marburg (bottom). Two projection methods were used: (1) Latitude/Longitude and (2) Optimal Mass Transport (OMT).

**
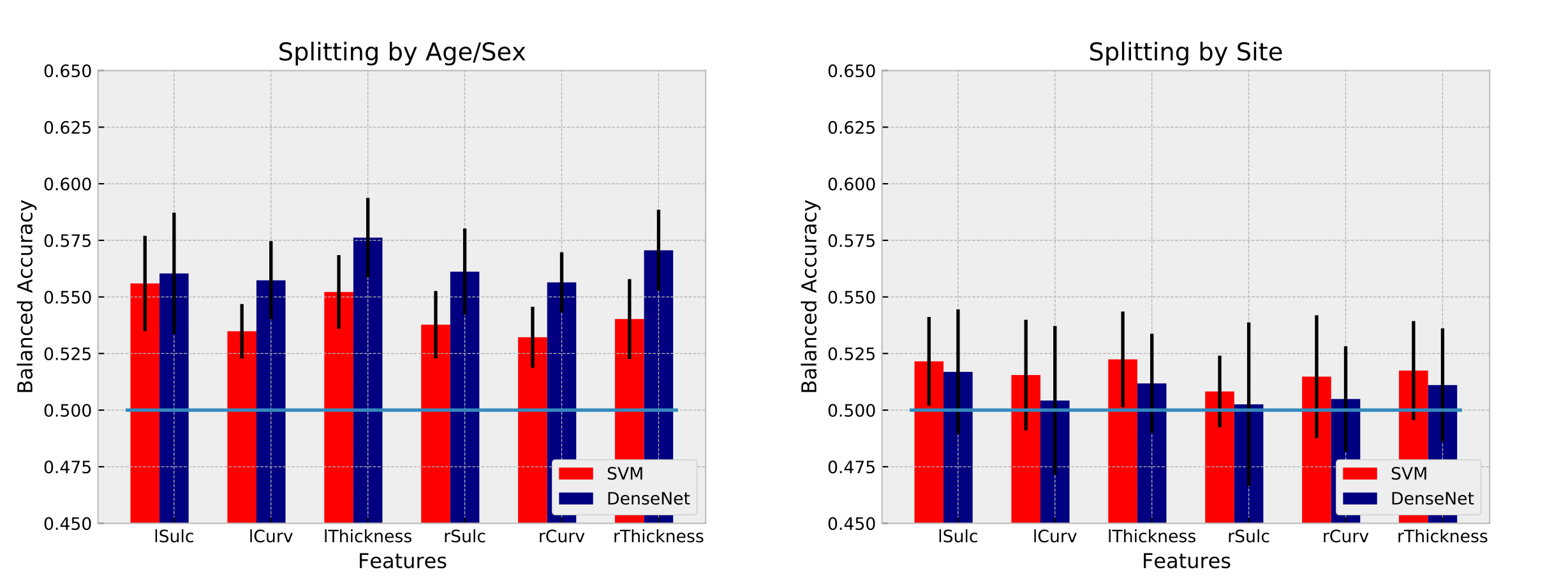
Supplementary Figure 3:** Major depressive disorder (MDD) vs. healthy control (HC) classification performance. Balanced accuracy of SVM and DenseNet classifiers was estimated separately for each hemisphere and feature type.

**
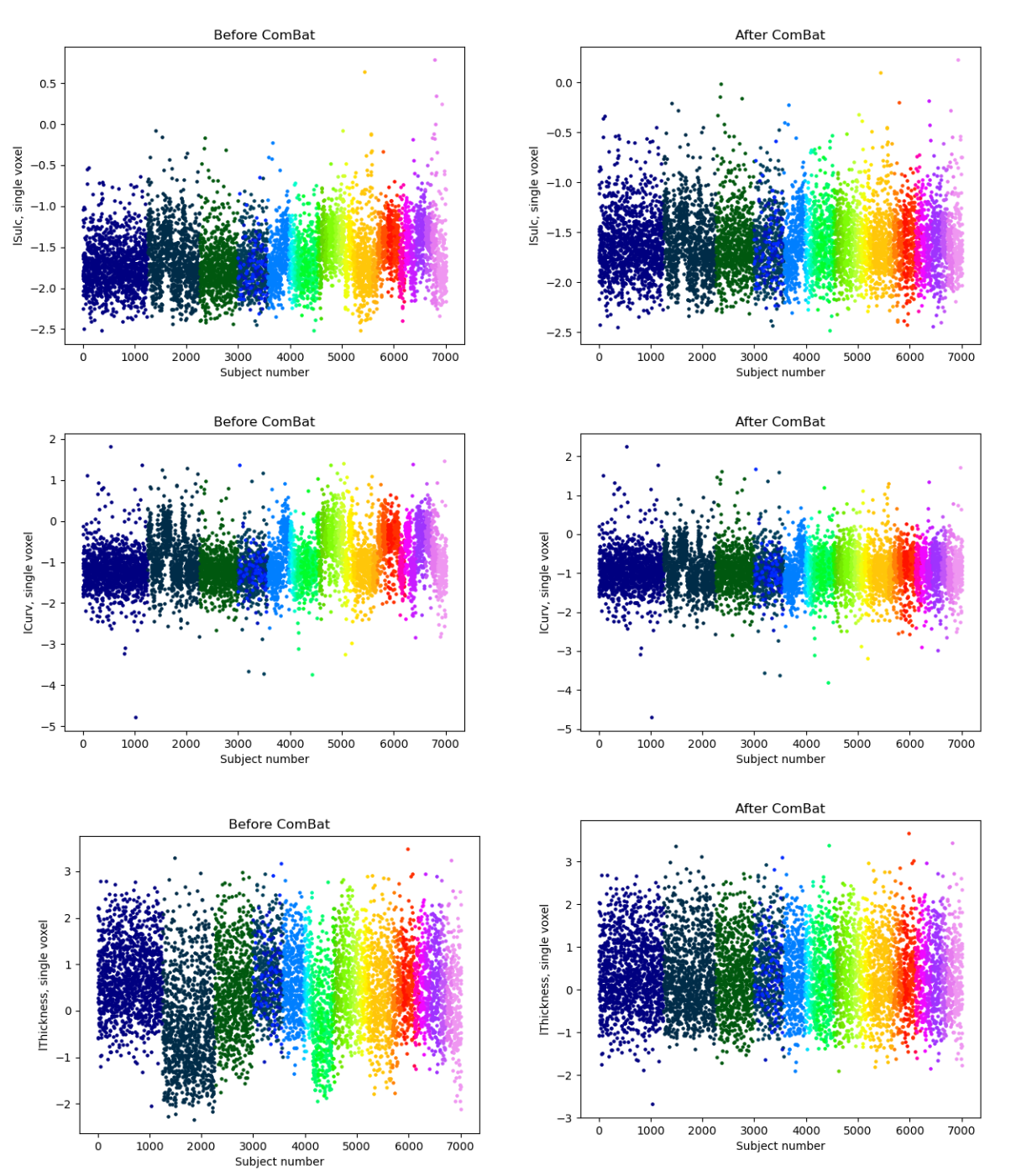
**

**Supplementary Figure 4:** Examples of ComBat harmonization across all data modalities. Colors indicate site affiliation, illustrating cohort-related variability before and after harmonization.
